# Artificial Intelligence Readiness, Perceptions, and Educational Needs Among Dental Students: A Cross‐Sectional Study

**DOI:** 10.1002/cre2.925

**Published:** 2024-07-05

**Authors:** Dalal Hammoudi Halat, Rula Shami, Alaa Daud, Waqas Sami, Abderrezzaq Soltani, Ahmed Malki

**Affiliations:** ^1^ Academic Quality Department QU Health, Qatar University Doha Qatar; ^2^ Department of Clinical Oral Health Sciences, College of Dental Medicine QU Health, Qatar University Doha Qatar; ^3^ Department of Pre‐Clinical Affairs, College of Nursing QU Health, Qatar University Doha Qatar

**Keywords:** artificial intelligence, dental students, health education

## Abstract

**Objectives:**

With Artificial Intelligence (AI) profoundly affecting education, ensuring that students in health disciplines are ready to embrace AI is essential for their future workforce integration. This study aims to explore dental students' readiness to use AI, perceptions about AI in health education and healthcare, and their AI‐related educational needs.

**Material and Methods:**

A cross‐sectional survey was conducted among dental students at the College of Dental Medicine, Qatar University. The survey assessed readiness for AI using the Medical Artificial Intelligence Readiness Scale (MAIRS). Students' perceptions of AI in healthcare and health education and their educational needs were also explored.

**Results:**

A total of 94 students responded to the survey. AI readiness scores were average (3.3 ± 0.64 out of 5); while participants appeared more ready for the vision and ethics domains of MAIRS, they showed less readiness regarding cognition and ability. Participants scored average on AI perceptions (3.35 ± 0.45 out of 5), with concerns regarding AI risks and disadvantages. They expressed a high need for knowledge and skills related to AI use in healthcare (84%), AI for health‐related research (81.9%), and AI in radiology and imaging procedures (79.8%). Student readiness had a significant correlation with AI perceptions and perceived level of AI knowledge.

**Conclusions:**

This is the first study in Qatar exploring dental students' AI readiness, perceptions, and educational needs regarding AI applications in education and healthcare. The perceived AI knowledge gaps could inform future curricular AI integration. Advancing AI skills and deepening AI comprehension can empower future dental professionals through anticipated advances in the AI‐driven healthcare landscape.

## 1 Introduction

Artificial Intelligence (AI) refers to systems, programs, and machines that can create defined rules, learn from experience, make decisions, and accomplish tasks often requiring human intelligence, thus modeling intelligent behavior with minimal human intervention (Islam, Laughter, and Sadid‐Zadeh 2022; Monett, Lewis, and Thórisson 2020). AI is leveraging a new generation of algorithms, the transformative influence of which is seen and forecasted across industry, social structures, science, the workplace, and the global economy (Dwivedi, Hughes, and Ismagilova 2021). Like other fields, the healthcare domain is potentially affected by AI technologies, influencing healthcare services, diagnostics, therapeutics, information processing, and research, in addition to complex ethical and regulatory frameworks (Väänänen et al. 2021; Rong et al. 2020; Goirand, Austin, and Clay‐Williams 2021). As AI plays a crucial role in healthcare, it is imperative for health education, as well as, to embrace advancements in this field (Wartman and Combs 2018).

The integration of AI in dental practice is witnessing a great surge, primarily in digital diagnostic methods, dental radiology, periodontology, orthodontics, esthetic dentistry, and oral cancer. This makes a pedagogical update inevitable for future dental professionals to master these tools (Thurzo et al. 2023; Carrillo‐Perez, Pecho, and Morales 2022; Ariji, Fukuda, and Kise 2019). In 2021, Yüzbaşıoğlu reported that current dental curricula do not sufficiently prepare students to incorporate AI technologies into their future practice (Yüzbaşıoğlu 2021), despite expected dental AI implications complementing human tasks and altering many aspects of dental clinical practice (Saghiri et al. 2023). Recently, Thurzo and Colleagues (Thurzo et al. 2023) called for extensive updates in all dental education areas, whether theoretical or clinical, so that curricula are adapted to AI in dental practice. Notably, embedding AI in dental education and instilling its pertinent competencies also involves the examination of potential ethical challenges, whereby it should be purposefully introduced, ensuring safe and ethical application for the benefit of both students and patients (Kim et al. 2023).

According to literature unleashing AI in education, new behavioral change due to AI is expected to be brought about in the academic landscape, including both learner and teacher adaptation to AI for enriching the educational experience (Chen, Chen, and Lin 2020), ability of AI to offer recommendations and feedback to learners (Luckin et al. 2016), and AI empowerment of students to recognize their learning activities and patterns, predict their intended learning outcomes, support them in course planning, and strategically regulate their learning (Luan, Geczy, and Lai 2020; Zawacki‐Richter et al. 2019). As such, in dental education, measuring students' readiness for change shall help in guiding educational adaptation, and is essential for fostering competent, knowledgeable, and skilled graduates (Al‐Maskari, Al Riyami, and Ghnimi 2022). An early assessment of readiness levels enables guidance that is tailored to students' unique characteristics, involving scrutinizing individual requirements and developing specific programs. Therefore, articulating AI readiness among dental students serves as a foundation for anticipated curricular modifications (Karaca, Çalışkan, and Demir 2021).

Beyond readiness, perceptions toward AI use in dentistry reflect skepticism, due to a lack of basic and continuing AI education, fear of replacing dentists, and anxiety regarding ethical issues (Roganović, Radenković, and Miličić 2023). Despite positive perceptions of how AI could revolutionize dental practice, some students expressed their worries over AI possibly replacing dental careers (Yüzbaşıoğlu 2021). Grasping perceptions of dental students toward AI remains essential in shaping dental education, addressing ethical considerations, fostering innovation, and ensuring seamless integration of AI from education into practice. An understanding of students' AI perceptions, knowledge gaps, and ethical apprehension is instrumental in informing curricular alterations (Civaner et al. 2022). As such, a needs assessment is a preliminary step to revise the curriculum, identify requirements for faculty development, and scrutinize learner status. Incorporation of AI competencies in dental education necessitates a rigorous needs assessment as an important facet of gathering input from stakeholders, thus making informed decisions for the benefit of students and the learning process (Leadbeatter and Bell 2018).

At Qatar University (QU), the College of Dental Medicine is a leader in providing comprehensive, integrated, and best‐practice dental education, aligned with QU's mission and nurturing competent students into future healthcare providers. AI is infused at several points across the dental curriculum, including a module about AI in dental research and also an elective course on AI in medicine. However, to our knowledge, the assessment of readiness, perceptions, and needs toward AI and its integration into dental curricula has been seldom addressed in the literature, and was not previously explored for dental students in Qatar. To address this knowledge gap, the aims of the current study were to assess QU dental students' readiness for the incorporation of AI in their education, to explore their perceptions of AI, and to assess their AI health educational needs.

## 2 Methods

### 2.1 Study Design

This was a cross‐sectional study conducted via an online survey using Blue platform and administered to students at the College of Dental Medicine at QU, during the Fall 2023 semester. The methodology followed the Checklist for Reporting Results of Internet E‐survey (CHERRIES) (Eysenbach 2004). Ethical approval for the study was obtained from the QU Institutional Review Board (reference number QU‐IRB 1957‐E/23).

### 2.2 Sample Size Estimation

With 152 registered dental students, 95% confidence interval, and a 5% margin of error, the minimum representative sample size was 110 (72% response rate). The survey was sent to all dental students in different cohorts to reduce any sampling bias. Alternatively, we targeted a reasonable response rate of 50% based on similar studies (Yüzbaşıoğlu 2021; Civaner et al. 2022).

### 2.3 Survey Instrument

The survey instrument was constructed upon a thorough review of literature on AI readiness, perceptions, and needs among health education students (Civaner et al. 2022; Wood, Ange, and Miller 2021; Ahmed, Bhinder, and Tariq 2022; Bisdas, Topriceanu, and Zakrzewska 2021), and was subject to revision versus available evidence. For content validity, the survey instrument was discussed among authors, and reviewed by faculty experts in AI. These included scholars specialized in AI in medicine, those with computer and engineering backgrounds who affiliate with the College of Dental Medicine, and those with extensive background of research in the dental field. Face validity was also conducted to check the survey instrument for clarity, readability, and time to completion. Accordingly, items were added, deleted, modified, and paraphrased to ensure clarity and allow participants to attempt questions within a reasonable time. The final version of the survey instrument underwent reliability testing for internal consistency using Cronbach's *α*.

The final survey instrument consisted of four sections. The first section addressed student demographics and an optional question about Grade Point Average (GPA). This section also inquired about participants' self‐evaluation of previous AI in health knowledge, previous AI training, and the nature and usefulness of this training, if any. The second section aimed at AI readiness evaluation using the 22‐item Medical Artificial Intelligence Readiness Scale (MAIRS) (Karaca, Çalışkan, and Demir 2021) including four domains that collectively ensure a holistic and responsible approach to adopting AI in medicine: cognition, ability, vision, and ethics. Cognition in the MAIRS pertains to understanding and knowledge of AI technologies, ensuring that participants grasp potential AI concepts, terminologies, and tools pertinent to healthcare. On the other hand, the ability domain focuses on technical skills and competencies for the effective implementation and utilization of AI solutions in healthcare delivery and patient care. The vision domain refers to the strategic foresight of AI regarding strengths, limitations, opportunities, and threats that its application poses to healthcare. The fourth, the last domain of ethics addresses the participant's ability to uphold moral and ethical considerations while applying AI, to assure patient rights, privacy, and fairness. Students were asked to rate their agreement with each statement using a 5‐point Likert scale. The third section included an assessment of students' perceptions of the possible influence of AI in health education and healthcare using 17 items rated by a 5‐point Likert scale. The rationale for selecting these statements was being able to portray how students envision their university education and later clinical practice with the introduction of AI. Of these statements, five targeted perceptions toward AI in health education, whereby participants were asked about their views regarding the influence and benefits of AI on learning and their expectations about AI in education. Also, seven statements targeted the perceived usefulness and reliability of AI in healthcare, such as medical decision‐making, medical errors, and patient confidence. The remaining five statements targeted perceived AI risks and disadvantages, mainly on the role of healthcare professionals, patient privacy, and bias. The fourth, final section assessed students' needs for AI in their curriculum, whereby 12 topics were included. The selection of these topics was inspired by previous surveys, (Civaner et al. 2022; Ahmed, Bhinder, and Tariq 2022) but mainly depended upon focused discussion among the authors regarding relevant health topics that should be included, such as AI applications in healthcare, diagnostics, prevention, research, and others. Participants were asked to rate the topics as not important, cannot tell, important, or very important in terms of inclusion in health education. The reliability of the final version of the survey instrument and the different scales was assessed using Cronbach's *α* at a cutoff of 0.7 for satisfactory internal consistency.

### 2.4 Data Collection

The survey was circulated between December 2023 and January 2024 to 152 dental students via email introducing the study scope and objectives and encouraging engagement. The survey was anonymous, participation was voluntary, and informed consent from participants was obtained online on the first page of the survey. Completing the next pages of the survey until its end and submission were considered an agreement to participate in the study.

### 2.5 Conceptual Framework

We developed a framework to visualize the relationship between the study variables using Directed Acyclic Graphs (DAGs) and the program DAGitty 3.0 (public domain) (Textor et al. 2016). DAGs help in identifying variables that should be considered in the evaluation of the effect of exposure on the outcome, as well as those for which adjustment is deemed inappropriate or unnecessary (Shrier and Platt 2008). The outcome was student readiness for AI in education; the exposure was perceptions of the possible influence of AI in health education and healthcare.

### 2.6 Statistical Analysis

#### 2.6.1 Scores Calculation

For each participant, we calculated scores for the 4 different domains of MAIRS and a total MAIRS score. Also, we calculated scores of perceptions of the possible influence of AI in health education, perceptions of usefulness and reliability of AI in healthcare, and perceptions of possible risks and disadvantages of AI, as well as an average perception score. Higher scores indicated higher readiness and more positive perceptions. Student ratings for each of the 12 AI topics were used to calculate a mean score for importance.

#### 2.6.2 Descriptive Analysis

Data normality was assessed using histograms and Kolmogorov–Smirnov test, and accordingly, we summarized continuous variables (age, scores of readiness, scores of perceptions) using means and standard deviations (for non‐skewed variables) and median and interquartile range (for skewed data). Categorical variables (gender, GPA, previous educational experience or training, self‐rating of AI knowledge, self‐rating of the usefulness of AI training, and the type of reported need for AI in education) were described using frequency distributions.

#### 2.6.3 Regression Analysis

Bivariate analysis was used to investigate the association between student readiness and each of socio‐demographic characteristics, and student perceptions scores using univariate regression. Then, we conducted multivariate analysis using a multiple linear regression model to explore the relationship between student readiness (dependent variable) and scores of perceptions (independent variable). Socio‐demographic variables were included in the model and were selected based on their potential to confound the relationship between student readiness and perceptions toward AI in education. For that purpose, we relied on DAGs and evidence from literature about factors with the potential to confound this association. In all analyses, a *p*‐value > 0.05 was considered a cutoff for statistical significance.

## 3 Results

A total of 94 dental students responded to the survey (61.84% response rate). The survey showed excellent internal consistency (Cronbach's *α* 0.93).

### 3.1 Student Background Characteristics

The majority of respondents were females (75%) and 53% reported a GPA of 3 or higher (out of 4). While the majority of respondents self‐evaluated their AI knowledge as basic, only 36.2% received AI educational experience or training, and those viewed training with various levels of usefulness, the majority considering it very useful (Table 1).

**Table 1:** Student socio‐demographic and academic characteristics (*N* = 94).

Baseline characteristics	Mean (SD)/*n* (%)
Age	20.1 (1.6)
Gender	
Female	70 (74.5)
Male	24 (25.5)
Year of study	
Year 1	16 (17.0)
Year 2	23 (24.5)
Year 3	27 (28.7)
Year 4	17 (18.1)
Year 5	11 (11.7)
Grade‐point average (GPA)	
Less than 2.5	2 (2.1)
2.5–2.9	11 (11.7)
3.0–3.49	13 (13.8)
3.5–3.79	18 (19.2)
3.8 or higher	19 (20.2)
Not applicable	13 (13.8)
*Missing*	18 (19.2)
How do you self‐evaluate your knowledge about AI use and applications in health	
I have no AI knowledge	14 (14.9)
I have basic AI knowledge	74 (78.7)
I have advanced AI knowledge	6 (6.4)
Previous educational experience or training in AI	
No educational/training experience	60 (63.8)
University‐based in‐person courses	26 (27.7)
University‐based online courses	1 (1.1)
Non‐university‐based courses or training	5 (5.3%)
Other educational activities	2 (2.1)
How do you rate the usefulness of the educational experience or training you received on AI?	
Of little usefulness	1 (1.1)
Of average usefulness	12 (12.8)
Very useful	18 (19.1)
Extremely useful	3 (3.2)
Not applicable	60 (63.8%)

### 3.2 Student Readiness

The results of AI readiness measured using the 22‐item MAIRS showed an overall readiness score of 3.30 ± 0.64 for an overall agreement rate of 5, indicating moderate readiness (Table 2). Among the MAIRS domains, the highest readiness was for vision (3.5 ± 0.95), followed by ethics (3.43 ± 0.92), then ability (3.31 ± 0.95), and the least for cognition (3.03 ± 0.88). In the first assessed MAIRS domain for cognition, notably, a major lack of readiness was in AI systems training (37.2% disagreement) and in workflows compatible with AI (31.9% disagreement). The highest readiness in the second MAIRS domain for ability was for valuable use of AI for education, service, and research (58.5% agreement), and in the second MAIRS domain for vision was the understanding of limitations, strengths, and weaknesses of AI (at least 54% agreement). In the fourth MAIRS domain for ethics, readiness was high for all assessed items and agreement was at least at 45%.

**Table 2:** Students' readiness toward applying AI in Health education and practice using the Medical Artificial Intelligence Readiness Scale (MAIRS).

Question item^a^	Strongly disagree	Disagree	Neutral	Agree	Strongly agree	Mean (SD)^b^
I can define the basic concepts of data science	5 (5.3%)	17 (18.1%)	43 (45.7%)	29 (30.9%)	0 (0%)	3.02 (0.84)
I can define the basic concepts of statistics	2 (2.1%)	7 (7.4%)	38 (40.4%)	44 (46.8%)	3 (3.2%)	3.41 (0.76)
I can explain how AI systems are trained	7 (7.4%)	28 (29.8%)	35 (37.2%)	24 (25.5%)	0 (0%)	2.81 (0.90)
I can define the basic concepts and terminology of AI	4 (4.3%)	24 (25.5%)	30 (31.9%)	35 (37.2%)	1 (1.1%)	3.05 (0.92)
I can properly analyze the data obtained by AI in healthcare	4 (4.3%)	23 (24.5%)	43 (45.7%)	24 (25.5%)	0 (0%)	2.93 (0.82)
I can differentiate the functions and features of AI‐related tools and applications	4 (4.3%)	21 (22.3%)	44 (46.8%)	23 (24.5%)	2 (2.1%)	2.98 (0.85)
I can organize workflows compatible with AI	6 (6.4%)	24 (25.5%)	39 (41.5%)	23 (24.5%)	2 (2.1%)	2.90 (0.91)
I can express the importance of data collection, analysis, evaluation, and safety; for the development of AI in healthcare	7 (7.4%)	15 (16.0%)	33 (35.1%)	33 (35.1%)	6 (6.4%)	3.17 (1.02)
*Overall score of cognition domain*						3.03 (0.88)
I can harness AI‐based information combined with my professional knowledge	7 (7.4%)	17 (18.1%)	34 (36.2%)	29 (30.9%)	7 (7.4%)	3.13 (1.03)
I can use AI technologies effectively and efficiently in healthcare delivery	5 (5.3%)	16 (17.0%)	39 (41.5%)	24 (25.5%)	10 (10.6%)	3.19 (1.01)
I can use artificial intelligence applications in accordance with their purpose	4 (4.3%)	9 (9.6%)	32 (34.0%)	41 (43.6%)	8 (8.5%)	3.42 (0.93)
I can access, evaluate, use, share, and create new knowledge using information and communication technologies	5 (5.3%)	15 (16.0%)	34 (36.2%)	34 (36.2%)	6 (6.4%)	3.22 (0.97)
I can explain how AI applications offer a solution to which problem in healthcare	5 (5.3%)	10 (10.6%)	38 (40.4%)	37 (40.4%)	4 (4.3%)	3.27 (0.90)
I find it valuable to use AI for education, service, and research purposes	2 (2.1%)	4 (4.3%)	33 (35.1%)	35 (37.2%)	20 (21.3%)	3.71 (0.92)
I can explain the AI applications used in healthcare services to the patient	3 (3.2%)	11 (11.7%)	39 (41.5%)	33 (35.1%)	8 (8.5%)	3.34 (0.91)
I can choose proper AI applications for the problems encountered in healthcare	2 (92.1%)	17 (18.1%)	39 (41.5%)	29 (30.9%)	7 (7.4%)	3.23 (0.90)
*Overall score of ability domain*						3.31 (0.95)
I can explain the limitations of AI technology	4 (4.3%)	7 (7.4%)	31 (33.0%)	40 (42.6%)	12 (12.8%)	3.52 (0.95)
I can explain the strengths and weaknesses of AI technology	2 (2.1%)	12 (12.8%)	29 (30.9%)	38 (40.4%)	13 (13.8%)	3.51 (0.95)
I can foresee the opportunities and threats that AI technology can create	2 (2.1%)	12 (12.8%)	31 (33.0%)	38 (40.4%)	11 (11.7%)	3.47 (0.93)
*Overall score of the vision domain*						3.50 (0.95)
I can use health data in accordance with legal and ethical norms	4 (4.3%)	6 (6.4%)	42 (44.7%)	31 (33.0%)	11 (11.7%)	3.41 (0.93)
I can conduct under ethical principles while using AI technologies	3 (3.2%)	7 (7.4%)	41 (43.6%)	32 (34.0%)	11 (11.7%)	3.44 (0.91)
I can follow legal regulations regarding the use of AI technologies in healthcare	4 (4.3%)	6 (6.4%)	39 (41.5%)	34 (36.2%)	11 (11.7%)	3.45 (0.93)
*Overall score of ethics domain*						3.43 (0.92)
*Overall readiness score*						3.30 (0.64)

^a^ Frequency distribution of question items is expressed as *n* (%).

^b^ Mean score and standard deviations of readiness items out of a possible score of 5, rated on a 1–5 Likert scale (1 = *strongly disagree*, 2 = *disagree*, 3 = *neutral*, 4 = *agree*, 5 = *strongly agree*). Higher scores indicate higher readiness.

### 3.3 Student Perceptions

The mean overall score of perceptions of the possible influence of AI in health education and healthcare was 3.35 ± 0.45 (Table 3). The most positive perceptions were about the influence of AI on health education (3.81 ± 0.70) followed by perceptions of perceived usefulness and reliability of AI in healthcare (3.54 ± 0.63). Most students indicated that AI should be included in university education (73.4%), and were willing to use it during their study (71.3%). Regarding the usefulness of AI for healthcare professionals, two‐thirds of students expressed agreement that AI facilitates access to patient data and allows them to make more accurate decisions. However, student responses reflected concerns regarding AI risks and disadvantages (2.70 ± 0.71). For example, over 50% agreed that AI can reduce human interaction in healthcare (59.6%), and that its potential bias can generate medical errors (51.1%).

**Table 3:** Students' perceptions on the possible influence of AI in health education and healthcare.

Question item^a^	Strongly disagree	Disagree	Neutral	Agree	Strongly agree	Mean (SD)^b^
Incorporating AI in health education will ease the learning process	1 (1.1%)	0 (0%)	31 (33.0%)	50 (53.2%)	12 (12.8%)	3.77 (0.71)
Using AI in health education will prepare me for future real‐life practice	1 (1.1%)	7 (7.4%)	22 (23.4%)	48 (51.1%)	16 (17.0%)	3.76 (0.86)
AI should be included in my health education during university study	1 (1.1%)	3 (3.2%)	21 (22.3%)	51 (54.3%)	18 (19.1%)	3.87 (0.79)
I am willing to use and benefit from AI during my university education	1 (1.1%)	2 (2.1%)	24 (25.5%)	48 (51.1%)	19 (20.2%)	3.87 (0.79)
At the end of my degree, I expect to have a better understanding of the use and applications of AI in healthcare	1 (1.1%)	4 (4.3%)	24 (25.5%)	47 (50.0%)	18 (19.1%)	3.82 (0.82)
*Overall score for perception subscale 1: Perceptions of AI in health education*						3.81 (0.70)
AI reduces errors in healthcare	1 (1.1%)	11 (11.7%)	37 (39.4%)	34 (36.2%)	11 (11.7%)	3.46 (0.88)
AI facilitates access to healthcare professionals to correct health information	2 (2.1%)	7 (7.4%)	36 (38.3%)	43 (45.7%)	6 (6.4%)	3.47 (0.81)
AI facilitates access to healthcare professionals to patient data	0 (0%)	6 (6.4%)	28 (29.8%)	51 (54.3%)	9 (9.6%)	3.67 (0.73)
AI allows healthcare professionals to make decisions that are more accurate	1 (1.1%)	4 (4.3%)	32 (34.0%)	47 (50.0%)	10 (10.6%)	3.65 (0.77)
AI allows patients to take better control of their health	0 (0%)	10 (10.6%)	28 (29.8%)	45 (47.9%)	11 (11.7%)	3.61 (0.83)
AI increases the confidence of patients in healthcare	2 (2.1%)	11 (11.7%)	33 (35.1%)	41 (43.6%)	7 (7.4%)	3.43 (0.87)
*Overall score for perception subscale 2: Perceived usefulness and reliability of AI in healthcare*						3.54 (0.63)
In the future, some of today's healthcare professions may be replaced by AI	8 (8.5%)	20 (21.3%)	28 (29.8%)	32 (34.0%)	6 (6.4%)	2.91 (1.07)
AI threatens to decrease the value of healthcare professions	9 (9.6%)	19 (20.2%)	27 (28.7%)	29 (30.9%)	10 (10.6%)	2.87 (1.14)
AI can reduce the trust between patients and healthcare professionals	3 (3.2%)	17 (18.1%)	35 (37.2%)	34 (36.2%)	5 (5.3%)	2.78 (0.91)
AI can reduce human interaction in healthcare	3 (3.2%)	11 (11.7%)	24 (25.5%)	42 (44.7%)	14 (14.9%)	2.44 (0.99)
AI can negatively affect the confidentiality of patient information	4 (4.3%)	16 (17.0%)	34 (36.2%)	32 (34.0%)	8 (8.5%)	2.74 (0.98)
AI potential bias can generate medical errors	0 (0%)	13 (13.8%)	33 (35.1%)	33 (35.1%)	15 (16.0%)	2.47 (0.92)
*Overall score for perception subscale 3: Possible risks and disadvantages of AI*						2.70 (0.71)
*Overall score of perceptions on the possible influence of AI in health education and healthcare*						3.35 (0.45)

^a^ Frequency distribution of question items is expressed as *n* (%).

^b^ Mean score and standard deviations of perception items out of a possible score of 5, rated on a 1–5 Likert scale (1 = *strongly disagree,* 2 = *disagree*, 3 = *neutral*, 4 = *agree*, 5 = *strongly agree*). Higher scores indicate more positive perceptions.

### 3.4 Student Ratings for Topics of Importance

The ratings of topics ranged between 2.8 and 3.12 out of 4. The most highly rated AI topics were “Knowledge and skills related to AI applications in healthcare,” “AI for health‐related research,” “AI in radiology and imaging procedures” with 84%, 81.9%, and 79.8% of participants, respectively, considering them important. The lowest rating was for topics of “AI in disease prevention” and “robotics in surgery” (Table 4).

**Table 4:** Student ratings of topics of importance to be included in AI education for health students.

Topic^a^	Not important	Cannot tell	Important	Very important	Mean (SD)^b^
Knowledge and skills related to AI applications in healthcare	4 (4.30%)	11 (11.70%)	63 (67.0%)	16 (17.0%)	2.97 (0.67)
AI in disease diagnosis	3 (3.20%)	22 (23.40%)	53 (56.40%)	16 (17.0%)	2.87 (0.72)
AI in disease monitoring	4 (4.30%)	21 (22.30%)	47 (50.0%)	22 (23.40%)	2.93 (0.79)
AI in genetics and genomics	7 (7.40%)	21 (22.30%)	41 (43.60%)	25 (26.60%)	2.89 (0.88)
AI in radiology and imaging procedures	4 (4.30%)	15 (16.0%)	48 (51.10%)	27 (28.70%)	3.04 (0.78)
AI in new drug development	5 (5.30%)	27 (28.70%)	37 (39.40%)	25 (26.60%)	2.87 (0.87)
AI and robotics in surgery	11 (11.70%)	18 (19.10%)	43 (45.70%)	22 (23.40%)	2.81 (0.93)
AI in surveillance and epidemic control	4 (4.30%)	26 (27.70%)	38 (40.40%)	26 (27.70%)	2.91 (0.85)
AI in mobile health applications for patient support	6 (6.40%)	16 (17.0%)	50 (53.20%0	22 (23.40%)	2.94 (0.81)
AI for reducing errors in healthcare	5 (5.30%)	25 (26.60%)	43 (45.70%)	21 (22.30%)	2.85 (0.829)
AI in disease prevention	4 (4.30%)	26 (27.70%)	49 (52.10%)	15 (16.0%)	2.80 (0.75)
AI for health‐related research	2 (2.10%)	15 (16.0%)	47 (50.00%)	30 (31.90%)	3.12 (0.74)

^a^ Frequency distribution of topics selected by students is expressed as *n* (%).

^b^ Mean score and standard deviations of topics' importance out of a possible score of 4, rated as (1) not important, (2) cannot tell, (3) important, or (4) very important. Higher scores indicate more topic importance for students.

### 3.5 Bivariate Analysis Using Univariate Regression

There was a strong positive relationship between scores of readiness and the overall score of (general) perceptions about AI in health education and healthcare (*p* < 0.001). Also, there was a strong positive relationship between the scores of readiness and the scores of perceived knowledge of AI (*p* < 0.001). Students who had any type of previous experience or training in AI had higher scores of readiness (*p* = 0.008). The nature of the training received by students was associated with scores of overall readiness (*p* = 0.03). There was no significant correlation between readiness scores and each of age, gender, year of study, GPA, or perceived usefulness of previous AI training (*p* values 0.63, 0.12, 0.39, 0.68, and 0.06, respectively).

### 3.6 Multiple Regression Analysis Results: Factors Associated With AI Readiness

**Figure 1:**
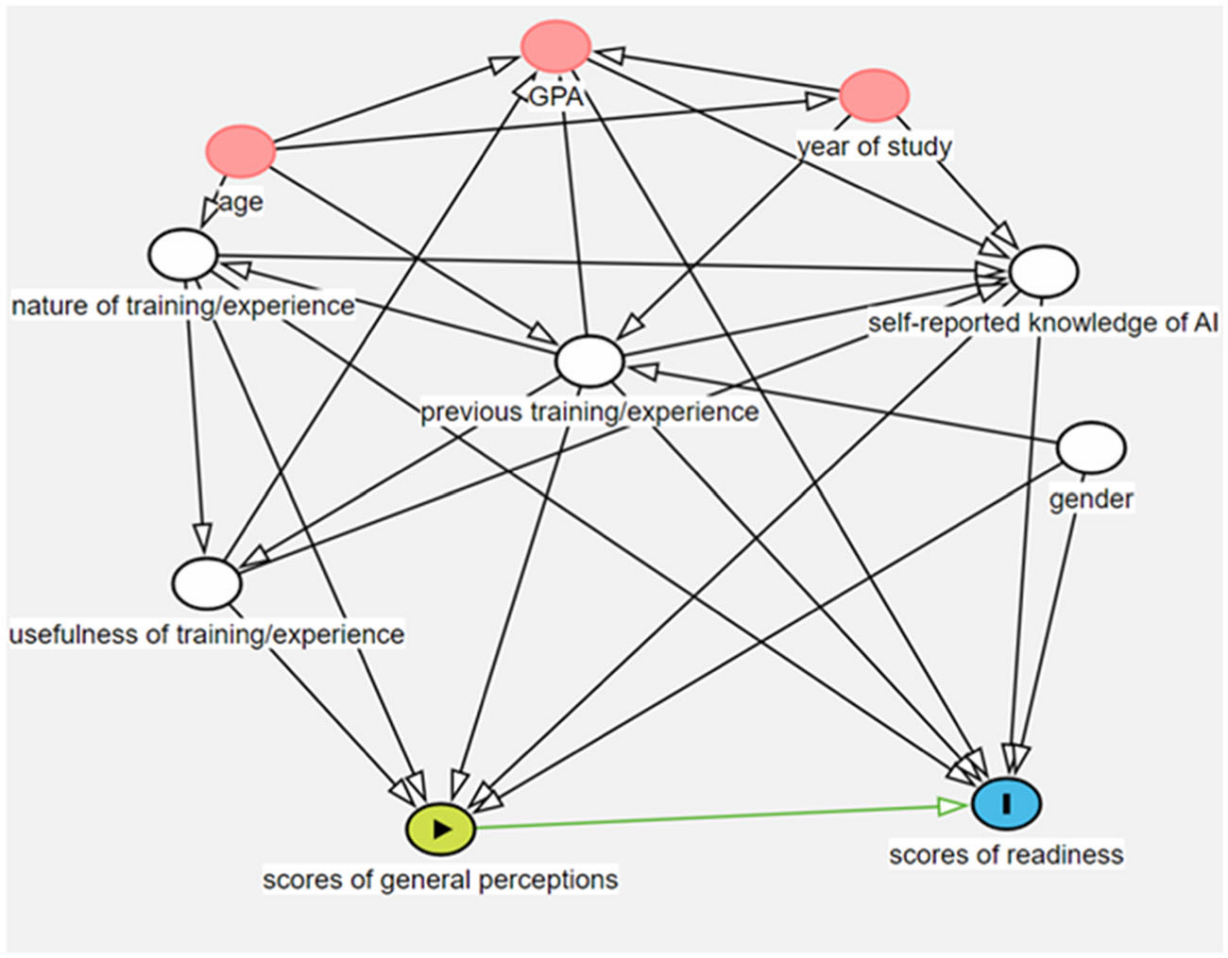
Directed Acyclic Graph of the association between students' readiness and general perceptions on the possible influence of AI in health education and healthcare. Green: exposure; blue: outcome; pink: unadjusted ancestor of exposure and outcome; white: adjusted ancestor of exposure and outcome.

A multiple regression model was built based on the minimum number of variables to be adjusted as indicated by the DAG in Figure 1 (five variables: gender, perceived level of AI knowledge, previous educational experience or training in AI, nature of training/education, perceived usefulness of AI training/education). Results showed a strong positive relationship between student readiness and each of the perceptions of the possible influence of AI in health education and healthcare and the perceived level of AI knowledge (Table 5). The readiness scores increased by 0.56 units with every unit increase in perception scores (*β* coefficient = 0.56; CI 0.33, 0.80). Readiness scores also increased with increasing levels of students' perceived AI knowledge (*β* coefficient = 0.48; CI 0.21, 0.75). The overall model was statistically significant and explained 41% of the variability in readiness scores (*R* ^2^ = 0.41, *F* = 10.08, *p* < 0.001).

**Table 5:** Association between student AI readiness scores and baseline characteristics of participants.

Variable	*β*‐coefficient	95% CI	*p* value
Scores of perceptions on the possible influence of AI in healthcare and education	0.56	(0.33, 0.80)	<0.001
Gender	−0.00	(−0.26, 0.24)	0.93
Perceived level of AI knowledge	0.48	(0.21, 0.75)	<0.001
Do you have any previous educational experience or training in AI?	0.54	(−0.26, 1.35)	0.18
Nature of previous AI training/education	−0.02	(−0.29, 0.24)	0.85
Perceived usefulness of AI training/education	0.13	(−0.13, 0.39)	0.32

## 4 Discussion

This study represents the first investigation regarding dental students' AI readiness, perceptions, and educational needs in Qatar. In dentistry, AI is expected to play a crucial role, making it compelling to understand student views as a part of the evidence for educational reforms into AI‐friendly curricula. According to the key findings of this study, participating students had moderate AI readiness, average perceptions about AI influence in health education and healthcare, and variable AI needs for their formal education.

Using MAIRS to assess readiness for AI, participants had an overall moderate readiness of about 3.3 out of 5. Lower readiness scores of 2.26–2.76 were depicted using the same instrument in a study from KSA among medical and dental professionals (Aboalshamat, Alhuzali, and Alalyani 2022). Our results also contrast with other findings among dental students and professionals, whereby a lack of AI knowledge was reported (Roganović, Radenković, and Miličić 2023). Among our participants, the highest readiness score was for the vision domain, followed by ethics, ability, and least for cognition, in contrast with studies reporting higher scores for cognitive and ability domains (Tung and Dong 2023; Xuan, Fahumida, and Al Nazir Hussain 2023). Our participants scored highest in the vision domain, which recounts to explaining limitations, strengths, and weaknesses related to medical AI, and anticipating opportunities and threats. Also, they perceived themselves relatively ready in the ethics domain, which relates to adherence to legal and ethical norms while using AI technologies in healthcare. This finding is remarkable, given the legal and ethical issues precipitated by newer digital technologies, and the risks of inaccuracy and data breaches with harmful consequences on patient care. This is especially important with the paucity of regulations to address legal and ethical issues of AI in healthcare settings (Naik, Hameed, and Shetty 2022). While results show an average score (3.43 out of 5) for the ethics domain of MAIRS, this should be interpreted carefully, with a focus on ethical AI dilemmas upon educating dental students, to better prepare them in this regard as future healthcare professionals.

However, as expressed by lower scores on the ability domain, our participants may be less prepared for competencies that allow choosing relevant AI applications, using them appropriately, and elucidating that to patients. Moreover, they appear least prepared with terminological knowledge of AI, logic of data science, and foundational AI principles, as reflected by scoring lowest in the cognitive domain. This was specifically noted in items related to AI technicalities like system training and workflow. As such, participants might not be sufficiently equipped with AI knowledge, comprehension, and practical use, suggesting that dental education institutions should prioritize these aspects when updating curricula, to better prepare students for AI in health. Currently, a cut‐off point for MAIRS has not been established in the literature, and therefore, a score for adequate readiness per se cannot be defined (Karaca, Çalışkan, and Demir 2021). It would be tempting to revisit readiness results when a pertinent MAIRS cut‐off has been defined by further research.

The perceptions of dental students toward the use of AI in health education and healthcare were also moderate, with higher agreement on statements pertaining to health education. For instance, at least 73% agreed that AI should be included in their health education; likewise, students were positive about the usefulness of AI for the learning process, preparation for practice, and understanding its use and applications as an outcome of their degree. These results resonate with previous calls to provide and introduce basic AI knowledge through education (Xuan, Fahumida, and Al Nazir Hussain 2023) and to innovate dental education with AI to enhance the pedagogical experience (Saghiri et al. 2023). Moreover, our participants perceived AI as useful and reliable in healthcare, with a score of 3.54 out of 5, reflecting beliefs about the positive influence of AI in accurate health information, patient data, reducing errors, and making correct decisions. Accordingly, students recognize AI's importance in health and acknowledge that university education and resources should embrace this new modality. Likewise, dental students previously reflected beliefs that AI would revolutionize dental practice, (Yüzbaşıoğlu 2021) necessitating the implementation of tailored health education and training to ensure that health professionals can leverage this new paradigm to improve health outcomes (Boillat, Nawaz, and Rivas 2022; Liu, Sawyer, and Luna 2022; Swed, Alibrahim, and Elkalagi 2022).

While the integration of AI in healthcare is progressively becoming pivotal, its discourse extends to ethical considerations, exploring potential biases and legal concerns (Upadhyay et al. 2023; Jeyaraman et al. 2023). In dentistry, the accelerating AI progress demands that dental education meticulously integrates AI into curricula, training graduates to use it ethically and responsibly (Kim et al. 2023). In this study, participants mostly agreed with the risks and disadvantages AI may pose to healthcare, especially reducing human interaction (59.6% agreement) and generating medical errors (51.1% agreement). They also expressed worries over trust, patient confidentiality, and professional value. These uncertainties have been previously reported, (Brundage, Avin, and Clark 2018; Finlayson et al. 2019) and call for efforts to regulate and protect patient data upon AI use in healthcare. However, only 40.4% of the participants agreed that AI may replace healthcare workers, a percentage close to those reported by Roganović and Colleagues (Roganović, Radenković, and Miličić 2023) and by Stewart and Colleagues (Stewart, Lu, and Gahungu 2023) among dental and medical students, respectively, but higher than reported in other surveys (Yüzbaşıoğlu 2021; Pinto Dos Santos, Giese, and Brodehl 2019; Gong, Nugent, and Guest 2019). In contrast to other professions, AI technology will encounter obstacles in substituting physicians or dentists. With existing challenges, such as AI's inability to counsel patients, establish trust, provide reassurance, and express empathy, AI will most likely support dentistry tasks rather than substitute the dentistry profession (Ngo, Nguyen, and vanSonnenberg 2021; Hilburg et al. 2020). Healthcare professionals should not simply adopt nor reject advancements in AI but rather contribute actively to conversations about AI that would impact their roles and the dynamics of their careers (Arnold 2021).

For AI needs assessment, we examined both, students' AI previous training, and their needs for specific health topics. While about 78% of participants claimed basic AI knowledge, about 64% did not receive previous AI training, and less than one‐third received structured AI education through university‐based courses. In their systematic review exploring AI in health education, and due to the lack of standardization in AI curricula and determined competencies, Sapci and Sapci (Sapci and Sapci 2020) recommend a specialized framework in health education to guide AI training. As such, students require foundational knowledge for AI in healthcare, requiring reframed educational programs to keep abreast of AI advances. The topic of AI in health‐related research was highly rated by participants, possibly triggered by their college exposure to professors who embed AI in their scholarly work, influencing their students. Also, the topic of AI in radiology and imaging scored high, given its relevance to dental practice and the current status of development (Heo, Kim, and Hwang 2021; Putra et al. 2022; Hung et al. 2020). The same was true for the topic of knowledge and skills related to AI applications in healthcare, indicating a willingness to gain competencies supporting future dentists as members of interdisciplinary teams, as previously reported (Civaner et al. 2022).

In the results of multivariate regression analysis, a strong positive relationship was found between students' AI readiness and perceptions of its influence in health education and healthcare. This indicates that students with positive expectations of AI value in health education and healthcare were more prepared for AI adoption in education and practice, revealing better readiness. Similarly, Chai and Colleagues (Chai, Wang, and Xu 2020) revealed that students' behavioral intentions toward learning AI correlate with perceived usefulness, social good, and attitudes toward its use. This carries implications that strengthening students' perceived control fosters their intention and readiness to learn AI, and accordingly embrace changes brought about by its use. Both AI readiness and perceived usefulness were linked to positive students' adoption of AI, as previously reported (Nouraldeen 2023).

Furthermore, a strong positive relationship existed between students' AI readiness and their perceived AI knowledge level, indicating that tech‐savvy respondents, who perceive themselves as more AI‐conversant, were more confident about AI benefits and ready for them. This association between students' AI readiness and self‐rated technological proficiency was recently described (Labrague et al. 2023), underscoring the importance of digital skills and technological command for ensuring optimal AI integration, while focusing on various learners' needs. Rainey, O'Regan and Matthew (2021).

The strength of this study lies in using a reliable instrument based on the validated MAIRS and including additional perception statements with good internal validity. The survey was created by a team with expertise in AI in medicine, health education, and quality improvements. We also addressed different student cohorts to obtain a comprehensive response set. However, this study has limitations; the cross‐sectional design skips gauging changes in readiness, perceptions, and needs over time. Also, with a single‐center design, findings cannot be generalized, albeit they give a snapshot of dental students' perceptions of AI and call for pertinent educational action steps. Furthermore, participants were limited to undergraduate dental students; the inclusion of doctoral students, practitioners, or specialized consultants may give a wider perspective on what AI in health education needs to accommodate for dentistry programs.

## 5 Conclusion

In conclusion, while dental students demonstrated overall average readiness and perceptions toward AI, notably, several AI knowledge gaps remain. AI will inevitably have major impacts on the next generation of dentists, and preparing the current and prospective workforce for AI integration in healthcare is imperative to safely and efficiently navigate a digital future. Identification of specific AI benefits, risks, and needs in dental education is helpful to fostering curricula and educational practices maximizing the usefulness of AI in dentistry, for both health education and personalized healthcare.

## Author Contributions

Dalal Hammoudi Halat and Ahmed Malki conceptualized this study idea. Dalal Hammoudi Halat did the literature review, obtained ethical approval, and wrote the initial manuscript draft. Dalal Hammoudi Halat, Waqas Sami, and Abderrezzaq Soltani designed the study survey. Rula Shami ran all statistical analyses and drafted the results. Alaa Daud acquired the data and assisted in manuscript drafting. Ahmed Malki was responsible for project administration and supervision. All authors critically revised the manuscript for intellectual content and approved the final version.

## Ethics Statement

Ethical approval for the study was obtained from the Qatar University Institutional Review Board (reference number QU‐IRB 1957‐E/23).

## Conflicts of Interest

The authors declare no conflicts of interest.

## Data Availability

The data supporting the findings of this study are available from corresponding authors on a reasonable request.
